# Comparative assessment of data obtained using empirical models for path loss predictions in a university campus environment

**DOI:** 10.1016/j.dib.2018.03.040

**Published:** 2018-03-16

**Authors:** Segun I. Popoola, Aderemi A. Atayero, Oluwafunso A. Popoola

**Affiliations:** Department of Electrical and Information Engineering, Covenant University, Ota, Nigeria

**Keywords:** Models, Forecasting, Path loss, Loss models, Radio propagation, Smart campus

## Abstract

Empirical models are most widely used for path loss predictions because they are simple, easy to use, and require less computational efficiency when compared to deterministic models. A number of empirical path loss models have been developed for efficient radio network planning and optimization in different types of propagation environments. However, data that prove the suitability of these models for path loss predictions in a typical university campus propagation environment are yet to be reported in the literature. In this data article, empirical prediction models are comparatively assessed using the path loss data measured and predicted for a university campus environment. Field measurement campaigns are conducted at 1800 MHz radio frequency to log the actual path losses along three major routes within the campus of Covenant University, Nigeria. Path loss values are computed along the three measurement routes based on four popular empirical path loss models (Okumura-Hata, COST 231, ECC-33, and Egli). Datasets containing measured and predicted path loss values are presented in a spreadsheet file, which is attached to this data article as supplementary material. Path loss prediction data of the empirical models are compared to those of the measured path loss using first-order statistics, boxplot representations, tables, and graphs. In addition, correlation analysis, Analysis of Variance (ANOVA), and multiple comparison post-hoc tests are performed. The prediction accuracies of the empirical models are evaluated based on Mean Absolute Error (MAE), Root Mean Squared Error (RMSE), and Standard Error Deviation (SED). In conclusion, the high-resolution path loss prediction datasets and the rich data exploration provided in this data article will help radio network engineers and academic researchers to determine the empirical model that is most suitable for path loss prediction in a typical university campus environment.

**Specifications table**

**Table:** 

Subject area	*Engineering*
More specific subject area	*Telecommunication Engineering*
Type of data	*Tables, graphs, figures, and spreadsheet file*
How data was acquired	*Field measurement campaigns are conducted at 1800* *MHz radio frequency to log the actual path losses along three major routes within the campus of Covenant University, Nigeria. Path loss values are computed along the three measurement routes based on four popular empirical path loss models (Okumura-Hata, COST 231, ECC-33, and Egli).*
Data format	*Raw, analyzed*
Experimental factors	*Field measurement campaigns were limited to areas covered by the lobes of the directional antennas of the 1800* *MHz base station antennas*
Experimental features	*Path loss prediction data of the empirical models are compared to those of the measured path loss using first-order statistics, boxplot representations, tables, and graphs. In addition, correlation analysis, Analysis of Variance (ANOVA), and multiple comparison post-hoc test are performed.*
Data source location	*Covenant University, Ota, Ogun State, Nigeria (Latitude 6°40'30.3"N, Longitude 3°09'46.3"E)*
Data accessibility	*Datasets containing measured and predicted path loss values are presented in a spreadsheet file, which is attached to this data article as*Supplementary material.

**Value of the data**

• Path loss data obtained using empirical prediction models are not often made available in regular research publications [1], [2], [3], [4]. This practically limits data reuse for required research reproducibility. In this data article, field measurement data and predicted path loss data are made freely available to the public domain. Also, the datasets are thoroughly described to facilitate further works among industry experts, radio network engineers, and academic researchers in this field of engineering.
• The suitability of empirical models for path loss predictions have been extensively evaluated for different scenarios and use cases within rural, suburban, and urban propagation environments [5], [6], [7], [8]. However, to the best of our knowledge, studies that focused on university campus environments are very limited. This data article focused on a smart campus use case in a bid to offer efficient Quality of Service (QoS) for smooth running of Internet of Things (IoT) applications within the university community [9].
• Over the years, several empirical models have been developed for path loss predictions [10], [11], [12]. However, data that prove the suitability of these models for path loss predictions in a typical university campus propagation environment are yet to be made available to the public. High-resolution path loss prediction datasets and rich data exploration are provided in this data article; and this information will help radio network engineers and academic researchers to determine the empirical model that is most suitable for path loss prediction in a typical university campus environment.
• Data exploration in this data article is supported with sufficient statistical analyses as done in [13], [14], [15], [16], [17], [18], [19].

## 1 Data

Path loss models are used to estimate radio network coverage and received signal strengths of transmitted electromagnetic waves at different points within a particular cell radius. The use of path loss models is a good alternative to carrying out actual measurements, which may be require much time and resources. There are three broad classes of path loss models namely: deterministic, semi-deterministic, and empirical [1], [3]. Empirical models are popularly used for path loss predictions because they are simple, easy to use, and require less computational efficiency when compared to deterministic models. A number of empirical path loss models have been developed for efficient radio network planning and optimization in different types of propagation environments. In this data article, field measurement data and predicted path loss data are made freely available to the public domain. Empirical prediction models are comparatively assessed using the path loss data measured and predicted for a university campus environment. Also, the datasets are thoroughly described to facilitate further works among industry experts, radio network engineers, and academic researchers in this field of engineering.

This data article focused on a smart campus use case in a bid to offer efficient Quality of Service (QoS) for smooth running of Internet of Things (IoT) applications within the university community. High-resolution path loss prediction datasets and rich data exploration are provided in this data article; and this information will help radio network engineers and academic researchers to determine the empirical model that is most suitable for path loss prediction in a typical university campus environment.

## 2 Experimental design, materials and methods

Actual path loss measurement data taken at the proposed propagation environment are required for objective comparative assessment of the prediction accuracy of empirical models in a university campus environment. Therefore, field measurement campaigns were conducted at 1800 MHz radio frequency under favourable climatic conditions and the actual path losses along three major routes (A, B, and C) within the campus of Covenant University, Ota, Nigeria (Latitude 6°40'30.3"N, Longitude 3°09'46.3"E) were recorded. The experimental design and setup consists of TEMS™ Investigation software developed by InfoVista®, Sony Ericsson® W995 mobile phones, Garmin Global Positioning System (GPS), and a Windows 7 Professional Operating System (OS) running on a laptop. The specifications of the Personal Computer (PC) is as follows: Intel® Core™ i5 Central Processing Unit (CPU) M520 @2.40 GHz processor; 4 GB Random Access Memory (RAM); 64-bit OS.

Path loss values were computed along the three measurement routes based on the mathematical equations of four popular empirical path loss models (Okumura-Hata, COST 231, ECC-33, and Egli) as given in [1], [3], [4]. All mathematical computations were performed using MATLAB 2017a produced by MathWorks Inc. Datasets containing measured and predicted path loss values are presented in a spreadsheet file, which is attached to this data article as Supplementary material. Path loss prediction data of the empirical models were compared to those of the measured path loss using first-order statistics, boxplot representations, tables, and graphs. In addition, correlation analysis, Analysis of Variance (ANOVA), and multiple comparison post-hoc test were performed. The prediction accuracies of the empirical models were evaluated based on Mean Absolute Error (MAE), Root Mean Squared Error (RMSE), and Standard Error Deviation (SED).

## 3 Data exploration

**Fig. 1:**
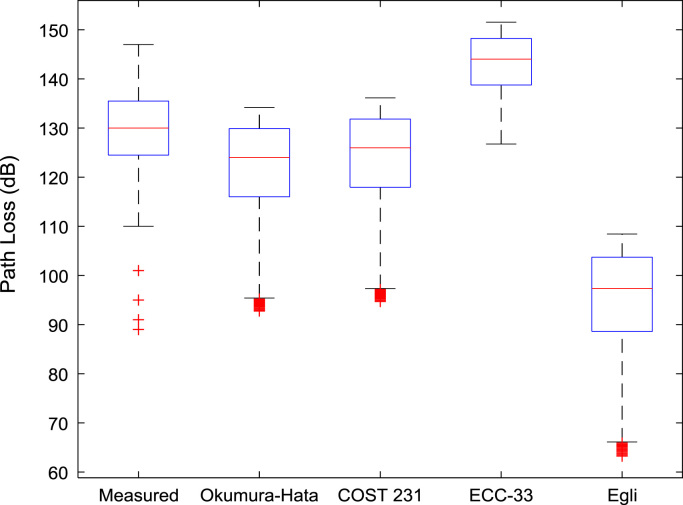
Boxplot representations of path loss predictions along measurement route A.

**Fig. 2:**
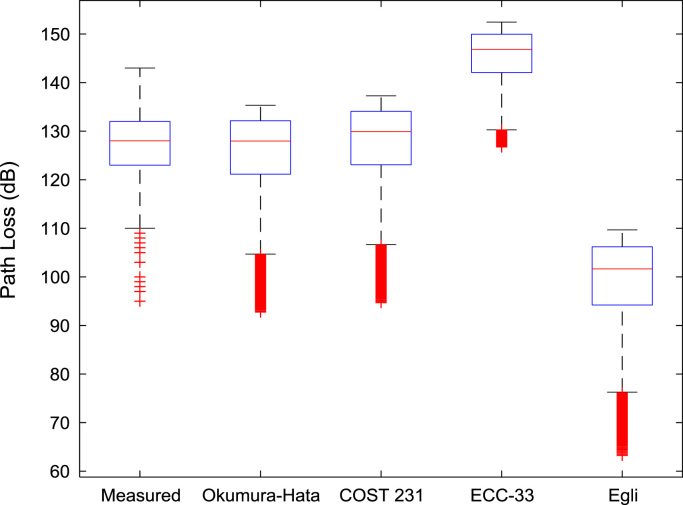
Boxplot representations of path loss predictions along measurement route B.

**Fig. 3:**
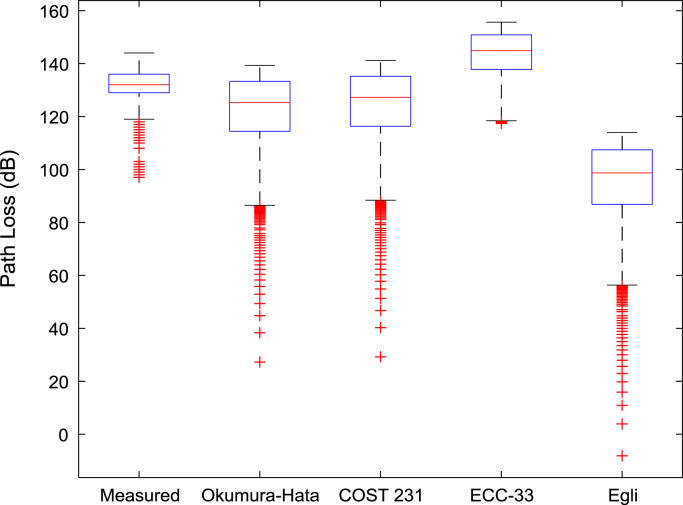
Boxplot representations of path loss predictions along measurement route C.

Table 1, Table 2, Table 3 present the descriptive statistics of measured path loss data and path loss values predicted by Okumura-Hata, COST 231, ECC-33, and Egli models for measurement routes A, B, and C respectively. The boxplot representations of the measured path loss data and the predicted path loss data for measurement routes A, B, and C are shown in Fig. 1, Fig. 2, Fig. 3 respectively.

**Table 1:** First-order statistics of path loss predictions along measurement route A.

	**Measured path loss (dB)**	**Okumura-hata model (dB)**	**COST 231 model (dB)**	**ECC-33 model (dB)**	**Egli model (dB)**
Mean	129.40	121.50	123.45	142.81	94.59
Median	130.00	124.04	125.99	144.04	97.36
Mode	129.00	92.77	94.71	126.76	63.25
Standard Deviation	8.30	10.64	10.64	6.68	11.61
Variance	68.91	113.23	113.23	44.57	134.68
Kurtosis	4.76	3.03	3.03	2.51	3.03
Skewness	−0.72	−0.95	−0.95	−0.69	−0.95
Range	58.00	41.42	41.43	24.79	45.18
Minimum	89.00	92.77	94.71	126.76	63.25
Maximum	147.00	134.19	136.14	151.55	108.43
Sample Size	496	496	496	496	496

**Table 2:** First-order statistics of path loss predictions along measurement route B.

	**Measured path loss (dB)**	**Okumura-hata model (dB)**	**COST 231 model (dB)**	**ECC-33 model (dB)**	**Egli model (dB)**
Mean	125.84	124.23	126.17	144.72	97.56
Median	128.00	127.97	129.91	146.83	101.64
Mode	126.00	92.77	94.71	126.76	63.25
Standard Deviation	9.44	11.09	11.09	6.96	12.09
Variance	89.12	122.99	122.99	48.48	146.29
Kurtosis	5.33	3.88	3.88	3.32	3.88
Skewness	−1.52	−1.39	−1.39	−1.16	−1.39
Range	48.00	42.57	42.57	25.69	46.43
Minimum	95.00	92.77	94.71	126.76	63.25
Maximum	143.00	135.34	137.28	152.45	109.68
Sample Size	547	547	547	547	547

**Table 3:** First-order statistics of path loss predictions along measurement route C.

	**Measured Path Loss (dB)**	**Okumura-Hata Model (dB)**	**COST 231 model (dB)**	**ECC-33 model (dB)**	**Egli model (dB)**
Mean	131.54	120.87	122.82	143.19	93.90
Median	132.00	125.29	127.24	144.91	98.72
Mode	132.00	27.28	29.23	117.39	-8.16
Standard Deviation	6.96	17.03	17.03	9.40	18.58
Variance	48.38	290.11	290.11	88.43	345.05
Kurtosis	9.00	6.61	6.61	2.81	6.61
Skewness	−1.88	−1.67	−1.67	−0.76	−1.67
Range	47.00	112.01	112.01	38.27	122.15
Minimum	97.00	27.28	29.23	117.39	-8.16
Maximum	144.00	139.29	141.24	155.66	113.99
Sample Size	773	773	773	773	773

**Fig. 4:**
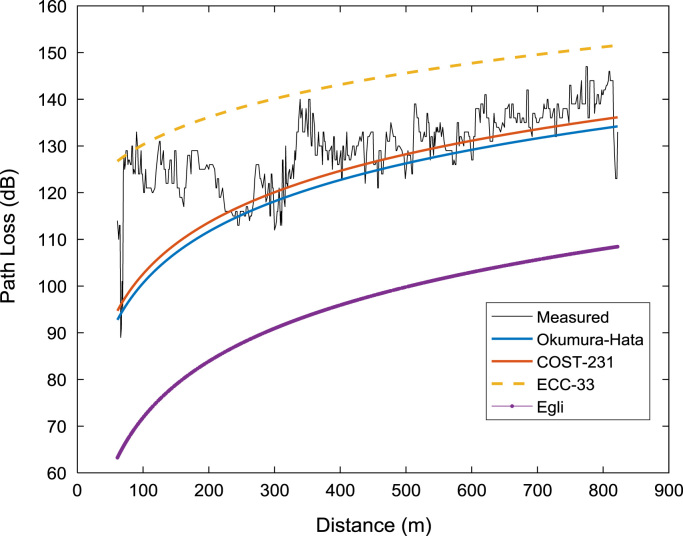
Path loss predictions against separation distance along measurement route A.

**Fig. 5:**
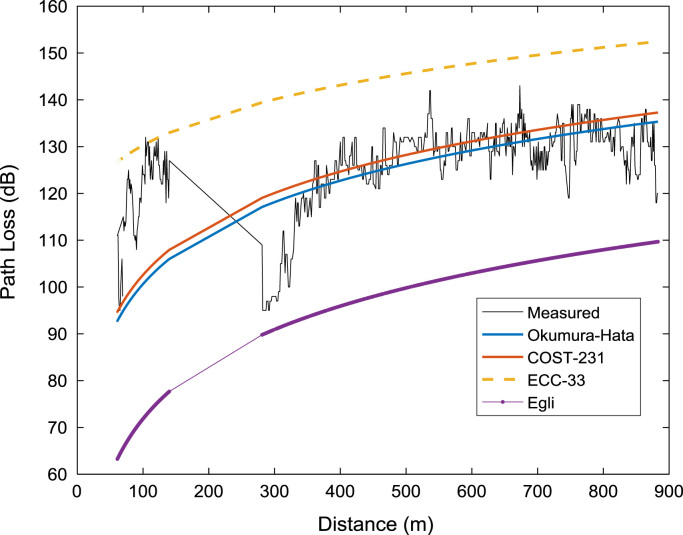
Path loss predictions against separation distance along measurement route B.

**Fig. 6:**
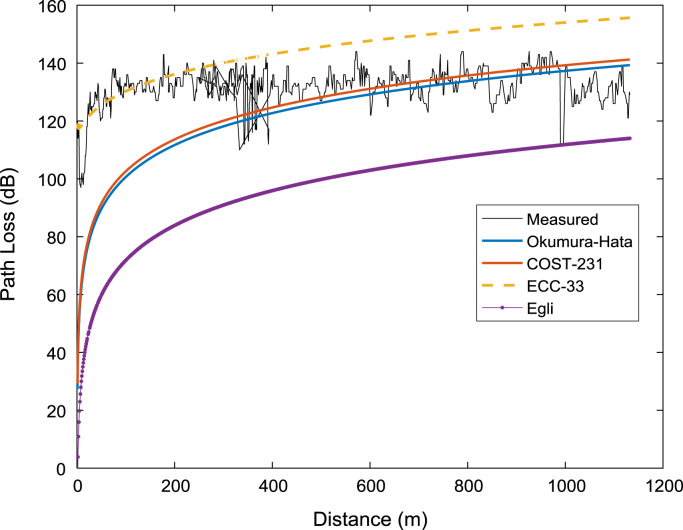
Path loss predictions against separation distance along measurement route C.

Signal path loss usually increase as the mobile receiver station moves further away from the transmitting base station. The relationships between the path loss datasets (measured and predicted) and the separation distance between the receiver and the transmitter for the three measurement routes (A, B, C) are depicted in the plots shown in Fig. 4, Fig. 5, Fig. 6. It is clear that the predictions of Okumura-Hata and COST 231 models are much closer to those of the actual measured data. However, the two models under-predicted the path loss values for distances below 200 m. On the other hand, ECC-33 model over-predicted the path loss values while Egli model under-predicted the path loss values throughout the distance range covered in this study.

**Fig. 7:**
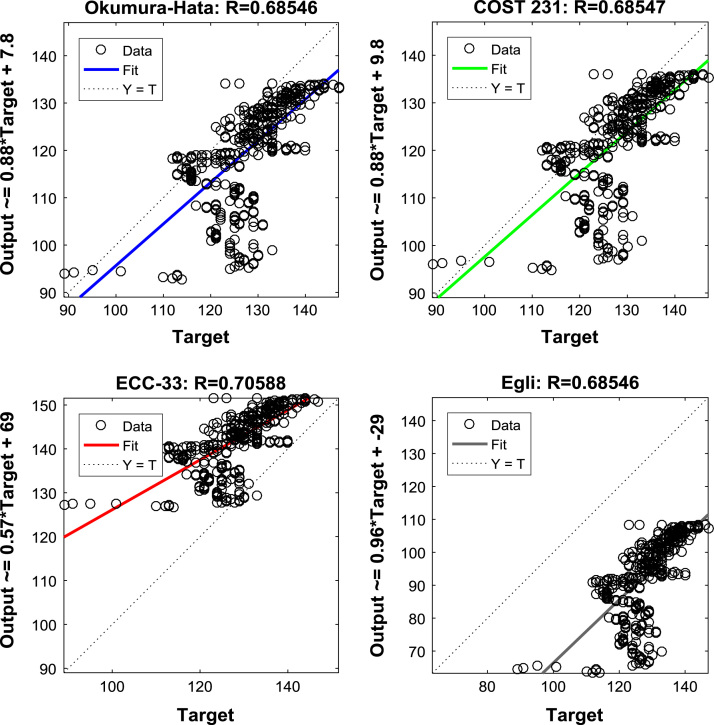
Regression Coefficients of the predictions of empirical models along measurement route A.

**Fig. 8:**
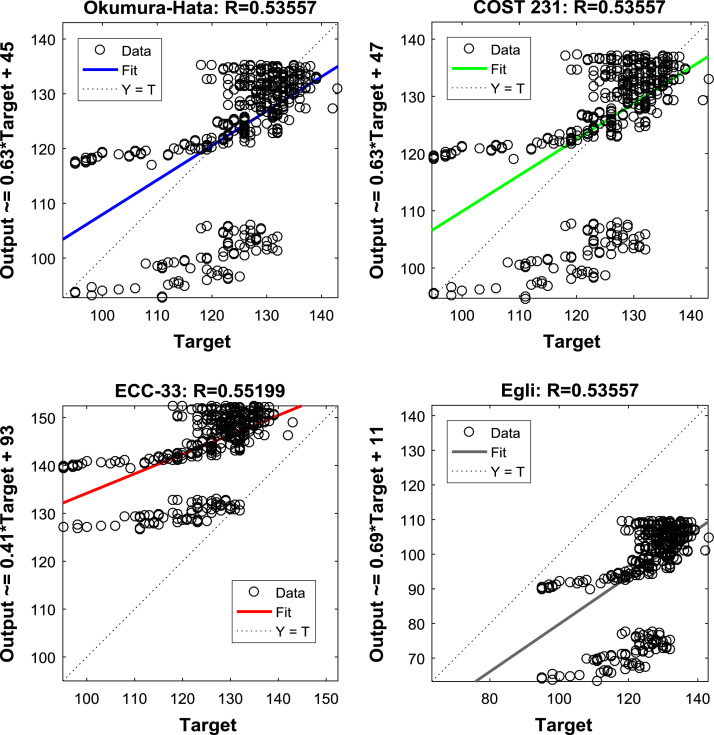
Regression Coefficients of the predictions of empirical models along measurement route B.

**Fig. 9:**
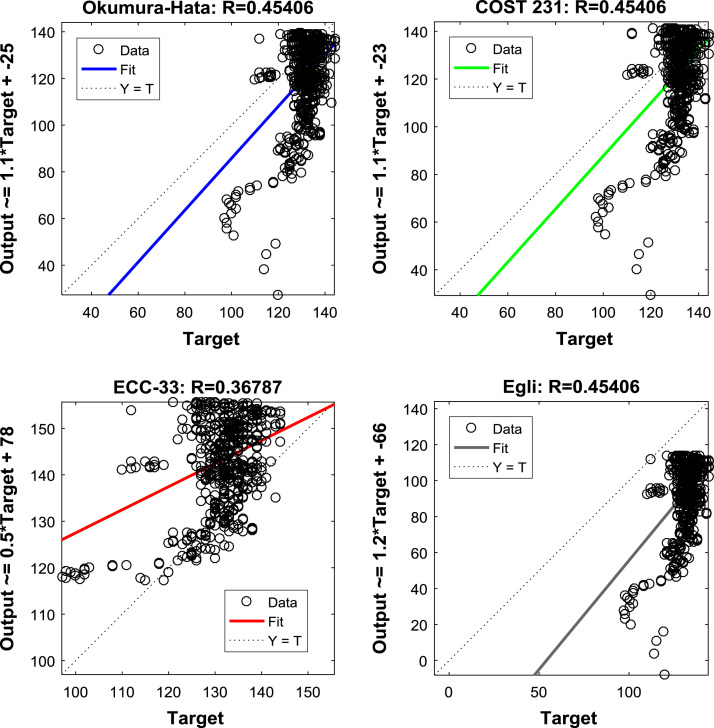
Regression Coefficients of the predictions of empirical models along measurement route C.

The regression equations and coefficients of the Okumura-Hata, COST 231, ECC-33, and Egli model prediction data, relative to the measured path loss data, are shown in Fig. 7, Fig. 8, Fig. 9. These information will help in understanding the relationships between the measured data and the predicted data. Further insights about the relationships can be gained from the results of correlation analyses presented in Table 4, Table 5, Table 6.

**Table 4:** Correlation coefficient matrix for predictions of empirical models along measurement route A.

	Measured	Okumura-Hata	COST 231	ECC-33	Egli
Measured	1.0000	0.6855	0.6855	0.7059	0.6855
Okumura-Hata	0.6855	1.0000	1.0000	0.9967	1.0000
COST 231	0.6855	1.0000	1.0000	0.9967	1.0000
ECC-33	0.7059	0.9967	0.9967	1.0000	0.9967
Egli	0.6855	1.0000	1.0000	0.9967	1.0000

**Table 5:** Correlation coefficient matrix for predictions of empirical models along measurement route B.

	Measured	Okumura-Hata	COST 231	ECC-33	Egli
Measured	1.0000	0.5356	0.5356	0.5520	0.5356
Okumura-Hata	0.5356	1.0000	1.0000	0.9970	1.0000
COST 231	0.5356	1.0000	1.0000	0.9970	1.0000
ECC-33	0.5520	0.9970	0.9970	1.0000	0.9970
Egli	0.5356	1.0000	1.0000	0.9970	1.0000

**Table 6:** Correlation coefficient matrix for predictions of empirical models along measurement route C.

	Measured	Okumura-Hata	COST 231	ECC-33	Egli
Measured	1.0000	0.4541	0.4541	0.3679	0.4541
Okumura-Hata	0.4541	1.0000	1.0000	0.9724	1.0000
COST 231	0.4541	1.0000	1.0000	0.9724	1.0000
ECC-33	0.3679	0.9724	0.9724	1.0000	0.9724
Egli	0.4541	1.0000	1.0000	0.9724	1.0000

**Fig. 10:**
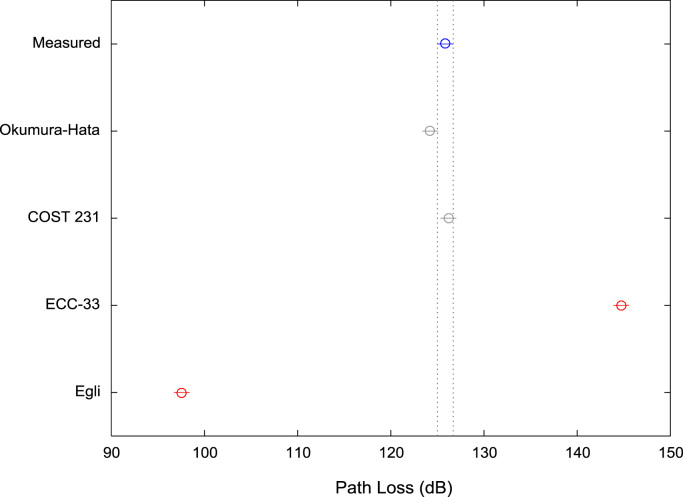
Graphical representation of post-hoc results along route A.

**Fig. 11:**
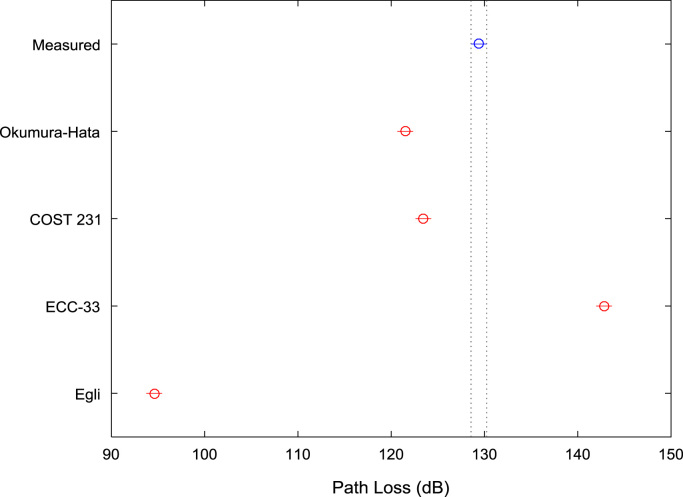
Graphical representation of post-hoc results along route B.

**Fig. 12:**
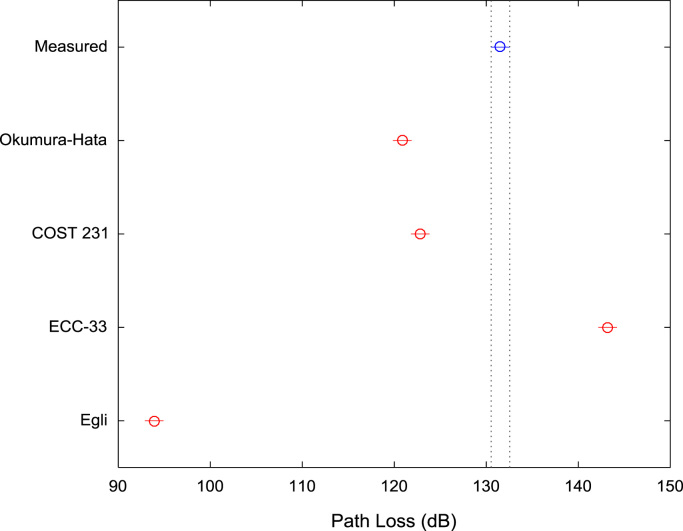
Graphical representation of post-hoc results along route C.

ANOVA and multiple comparison post-hoc tests were performed to understand whether the differences in the mean path losses obtained using the four models are significant. If so, the multiple comparison post-hoc test shows the extent to which the mean path losses differ from one another. The test results of the ANOVA test for path loss predictions along measurement route A, B, and C are presented in Table 7, Table 8, Table 9. Comparing the prediction outputs of Okumura-Hata, COST 231, ECC-33, and Egli models with one another, the lower limits for 95% confidence intervals, mean difference, upper limits for 95% confidence intervals, and the p-values obtained for measurement routes A, B, and C are presented in Table 10, Table 11, Table 12. The results are further depicted by the plots shown in Fig. 10, Fig. 11, Fig. 12.

**Table 7:** ANOVA test results for path loss predictions along measurement route A.

Source of variation	Sum of squares	Degree of freedom	Mean squares	F statistic	Prob>F
Columns	615539.2	4	153884.8	1621.12	0
Error	234939.4	2475	94.9		
Total	850478.6	2479

**Table 8:** ANOVA test results for path loss predictions along measurement route B.

Source of variation	Sum of squares	Degree of freedom	Mean squares	F statistic	Prob>F
Columns	621404.3	4	155351.1	1465.92	0
Error	289311.8	2730	106		
Total	910716.1	2734

**Table 9:** ANOVA test results for path loss predictions along measurement route C.

Source of variation	Sum of squares	Degree of freedom	Mean squares	F statistic	Prob>F
Columns	1028370	4	257092.5	1210.31	0
Error	819935	3860	212.4		
Total	1848305	3864

**Table 10:** Multiple comparison post-hoc test results for predictions along route A.

**Groups Compared**		**Lower limits for 95% confidence intervals**	**Mean difference**	**Upper limits for 95% confidence intervals**	***p*****-value**
Measured	Okumura-Hata	6.2093	7.8970	9.5846	0.0000
Measured	COST 231	4.2632	5.9508	7.6384	0.0000
Measured	ECC-33	−15.1048	−13.4172	−11.7296	0.0000
Measured	Egli	33.1198	34.8074	36.4950	0.0000
Okumura-Hata	COST 231	−3.6338	−1.9462	−0.2586	0.0143
Okumura-Hata	ECC-33	−23.0018	−21.3142	−19.6265	0.0000
Okumura-Hata	Egli	25.2228	26.9104	28.5980	0.0000
COST 231	ECC-33	−21.0556	−19.3680	−17.6804	0.0000
COST 231	Egli	27.1690	28.8566	30.5442	0.0000
ECC-33	Egli	46.5370	48.2246	49.9122	0.0000

**Table 11:** Multiple comparison post-hoc test results for predictions along route B.

**Groups Compared**		**Lower limits for 95% confidence intervals**	**Mean difference**	**Upper limits for 95% confidence intervals**	***p*****-value**
Measured	Okumura-Hata	−0.0785	1.6194	3.3174	0.0700
Measured	COST 231	−2.0244	−0.3264	1.3716	0.9849
Measured	ECC-33	−20.5728	−18.8748	−17.1769	0.0000
Measured	Egli	26.5852	28.2832	29.9811	0.0000
Okumura-Hata	COST 231	−3.6438	−1.9459	−0.2479	0.0153
Okumura-Hata	ECC-33	−22.1923	−20.4943	−18.7963	0.0000
Okumura-Hata	Egli	24.9658	26.6637	28.3617	0.0000
COST 231	ECC-33	−20.2464	−18.5484	−16.8504	0.0000
COST 231	Egli	26.9116	28.6096	30.3076	0.0000
ECC-33	Egli	45.4600	47.1580	48.8560	0.0000

**Table 12:** Multiple comparison post-hoc test results for predictions along route C.

**Groups Compared**		**Lower limits for 95% confidence intervals**	**Mean difference**	**Upper limits for 95% confidence intervals**	***p*****-value**
Measured	Okumura-Hata	8.6480	10.6702	12.6925	0.0000
Measured	COST 231	6.7021	8.7243	10.7466	0.0000
Measured	ECC-33	−13.6718	−11.6496	−9.6274	0.0000
Measured	Egli	35.6155	37.6377	39.6600	0.0000
Okumura-Hata	COST 231	−3.9681	−1.9459	0.0763	0.0659
Okumura-Hata	ECC-33	−24.3421	−22.3198	−20.2976	0.0000
Okumura-Hata	Egli	24.9453	26.9675	28.9897	0.0000
COST 231	ECC-33	−22.3962	−20.3739	−18.3517	0.0000
COST 231	Egli	26.8912	28.9134	30.9356	0.0000
ECC-33	Egli	47.2651	49.2873	51.3096	0.0000

In conclusion, the prediction accuracies of the empirical models are evaluated based on MAE, RMSE, and SED. The values of the performance metrics for measurement routes A, B, and C are presented in Table 13, Table 14, Table 15. In essence, the empirical evidence and statistical analyses provided in this data article will help radio network engineers and academic researchers to determine the empirical model that is most suitable for path loss prediction in a typical university campus environment.

**Table 13:** Statistical evaluation of predictions of empirical models along route A.

	Okumura-Hata	COST 231	ECC-33	Egli
Mean Absolute Error	8.4785	7.0738	13.4511	34.8074
Mean Squared Error	123.2816	96.3332	215.1928	1282.9000
Root Mean Squared Error	11.10322	9.814948	14.66945	35.81759
Standard Error Deviation	7.8130	7.8131	5.9366	8.4568

**Table 14:** Statistical evaluation of predictions of empirical models along route B.

	Okumura-Hata	COST 231	ECC-33	Egli
Mean absolute error	6.9801	7.0093	18.8815	28.2832
Mean squared error	102.4099	99.8952	421.1782	912.8365
Root mean squared error	10.11978	9.994759	20.52263	30.21318
Standard error deviation	9.9985	9.9986	8.0646	10.6351

**Table 15:** Statistical evaluation of predictions of empirical models along route C.

	Okumura-Hata	COST 231	ECC-33	Egli
Mean absolute error	13.7923	12.8628	12.3939	37.6377
Mean squared error	344.4628	306.7217	224.2870	1692.3000
Root mean squared error	18.55971	17.51347	14.97621	41.13757
Standard error deviation	15.1956	15.1956	9.4175	16.6162
